# A Rare Case of Isolated Pulmonary Cryptococcoma in an Immunocompromised Host

**DOI:** 10.7759/cureus.7748

**Published:** 2020-04-20

**Authors:** Jana Dbaibou, Diane L Levine

**Affiliations:** 1 Internal Medicine, Wayne State University, Detroit Medical Center, Detroit, USA

**Keywords:** cryptococcosis, hiv, cryptococcoma, immunocompromised, aids, pulmonary cryptococcoma, pulmonary cryptococcosis, disseminated disease, dissemination, localized

## Abstract

Cryptococcosis is an opportunistic fungal infection found in both immunocompromised and non-immunocompromised patients; however, it is particularly prevalent in those with Human Immunodeficiency Virus/Acquired Immunodeficiency Syndrome (HIV/AIDS). Patients with isolated pulmonary cryptococcosis can present with heterogeneous symptoms. The rarity of this entity makes it difficult to recognize and diagnose. We present a case of a 54-year-old female with poorly controlled HIV and seizure disorder, who presented with suspected seizures. Her CD4 count was 7. Due to fever and headache, cryptococcal meningitis was suspected, and she was empirically started on liposomal amphotericin and flucytosine. Computed tomography (CT) of the head was negative for any acute intracranial process. Serum cryptococcal antigen was positive; however cerebrospinal fluid (CSF) studies from lumbar puncture (LP) were entirely negative, including CSF cryptococcal antigen. CT thorax demonstrated interval development of two solid pulmonary nodules in the right upper lobe (RUL). There was no other evidence of disseminated cryptococcal disease. CT-guided biopsy of the larger RUL was compatible with Cryptococcus species. Fungal cultures of sputum and blood were negative. The patient improved, and therapy was de-escalated from liposomal amphotericin and flucytosine to oral fluconazole, with a plan to complete a six- to twelve-month course of therapy. This case illustrates that in rare cases, Cryptococcal disease may still be localized despite having a positive serum Cryptococcal antigen. It also emphasizes the importance of a thorough investigation with multimodal diagnostic tools to evaluate for disseminated Cryptococcal disease, especially in those with a history of immunocompromise.

## Introduction

Cryptococcosis is an important opportunistic fungal infection that is usually diagnosed in immunocompromised patients, especially those with Human Immunodeficiency Virus/Acquired Immunodeficiency Syndrome (HIV/AIDS). It is also seen in patients with no history of immunocompromise. Pulmonary cryptococcosis is a rare clinical entity that may present as pulmonary nodules, pneumonia, or even acute respiratory distress syndrome (ARDS). The method of infection is by inhalation of cryptococcal spores from the environment, classically bird droppings or contaminated dust. The spectrum of symptoms in pulmonary cryptococcosis depends on the host’s defenses. In patients with severe immunocompromise, this condition is usually associated with other manifestations of disseminated disease, commonly involving the central nervous system (CNS), skin, and even bone [1]. From our literature review, there have been fewer than 40 reported cases where cryptococcosis presented as an isolated pulmonary disease in immunocompromised individuals. We present a case in which a patient with HIV/AIDS, not on combined antiretroviral therapy (cART) therapy, developed isolated cryptococcal pulmonary nodules in the absence of any other signs or symptoms of disseminated disease.

## Case presentation

This is a 54-year-old homeless female with a medical history notable for poorly controlled HIV, and seizure disorder. She presented to the emergency department for suspected seizure activity. Per the patient, she experienced lower extremity weakness and mouth foaming of a few minutes duration while walking into a store. The patient stated she had been off of her ART for 6 months. She also complained of subjective fever, chills, cough, yellowish sputum production, and weight loss. She was found to have an absolute CD4 count of 7 (2%). There was no leukocytosis. Her initial chest x-ray (CXR) revealed no consolidation or infiltrates. Urine legionella and streptococcus pneumoniae antigen testing was negative. Blood culture, respiratory panel, and respiratory culture were negative. The patient was reinitiated on cART regimen of emtricitabine/tenofovir alafenamide (FTC/TAF) and dolutegravir (DTG), in addition to *Mycobacterium avium* complex (MAC) and *Pneumocystis *pneumonia (PCP) prophylaxis. The patient was treated for her acute on chronic seizure disorder, and as she was improving, discharge planning was pursued.

**Figure 1:**
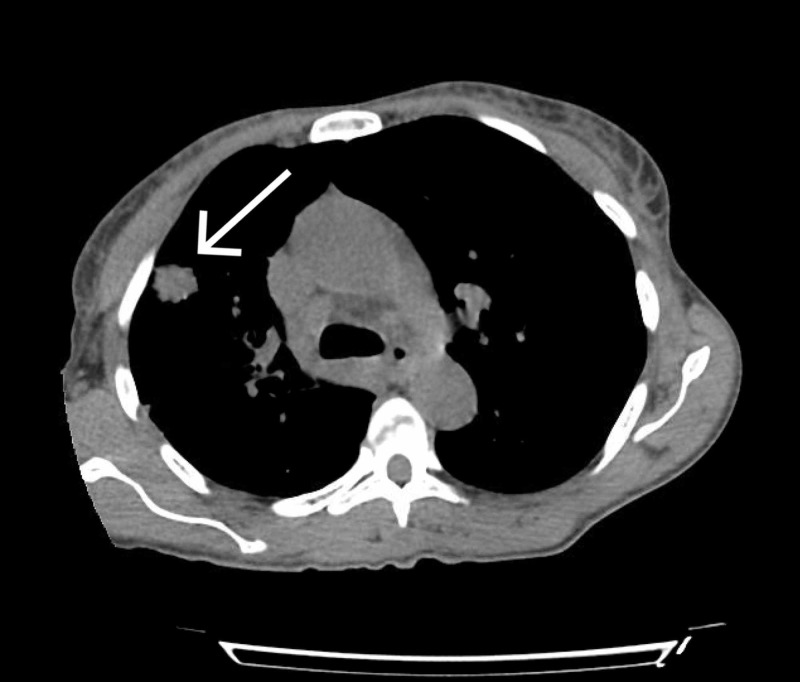
CT thorax demonstrating right upper lobe nodule measuring 1.9 x 1.6 cm CT, computed tomography

**Figure 2:**
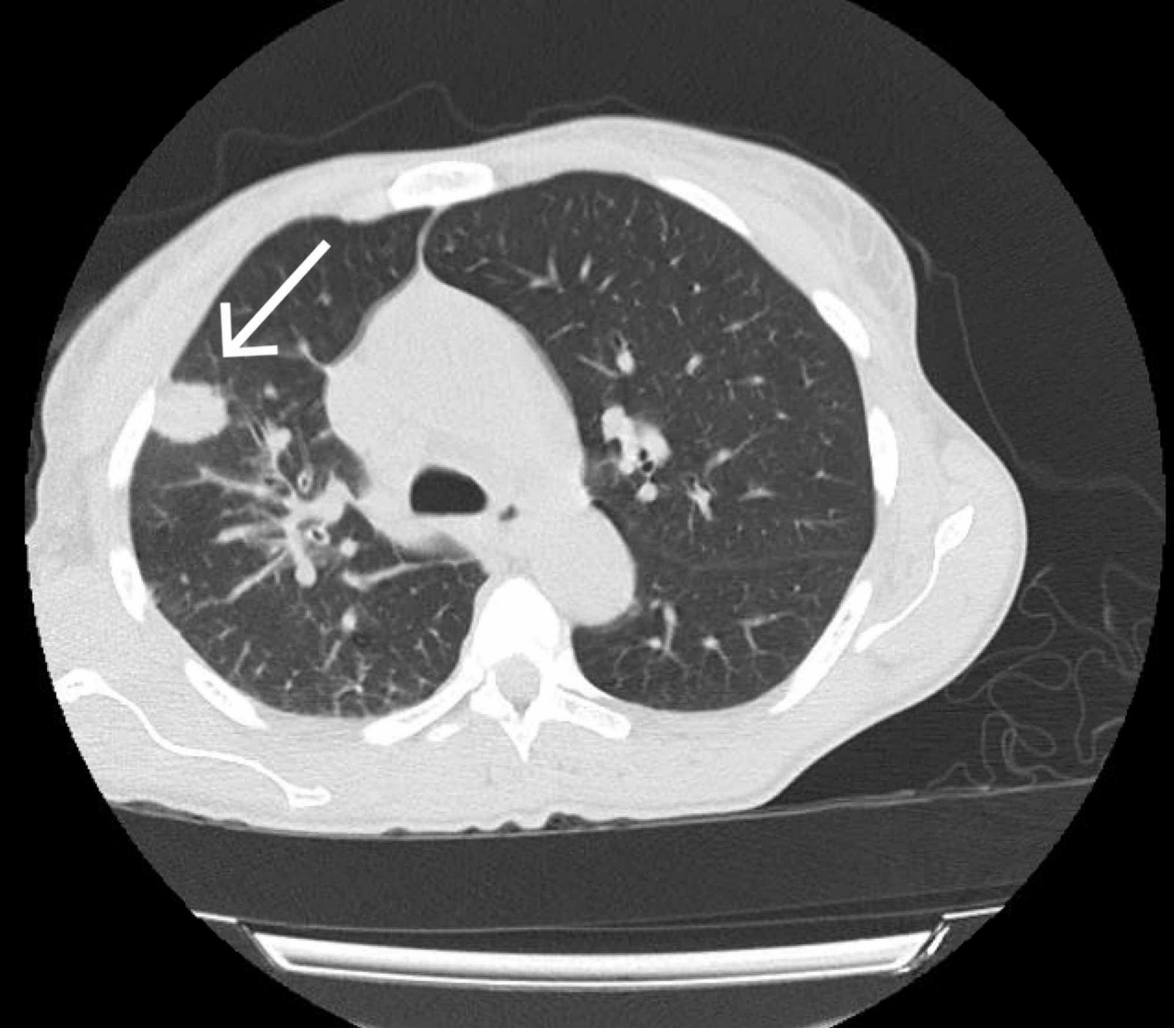
CT thorax demonstrating right upper lobe nodule measuring 1.9 x 1.6 cm CT, computed tomography

On day 12 of admission, the patient developed a fever of 39.4 Celsius. She continued to endorse cough and sputum production, in addition to a new frontal headache. Repeat CXR demonstrated a nonspecific ill-defined opacity in the right lung concerning early pneumonia. Given the patient’s advanced HIV status and active symptoms, there was a low threshold for assessment of tuberculosis (TB) and cryptococcal meningitis. She was placed on airborne precautions pending TB testing. Computed tomography (CT) of the head was obtained, demonstrating no evidence of any acute intracranial process. Despite the negative CT head imaging, she was empirically started on liposomal amphotericin and flucytosine due to high concern for cryptococcal meningitis. A lumbar puncture (LP) was planned; however, the patient initially refused. Serum cryptococcal antigen was sent, which required several days to result. CT thorax was obtained and demonstrated interval development of two solid pulmonary nodules in the right upper lobe (RUL), measuring 1.9 x 1.6 cm (Figures 1 and 2) and 0.5 x 0.5 cm, as well as right hilar lymphadenopathy. These findings were made in comparison to a CT thorax two years prior which had not shown any nodules or lymphadenopathy.

Three acid-fast bacilli (AFB) sputum stains were negative. Serum beta-D-glucan, *Blastomyces*, and *Histoplasma* antigens were sent and found to be negative. Urine antigen testing was not performed. Serum cryptococcal antigen testing was positive with a low titer of 1:16. Patient subsequently agreed to have the LP done, which revealed an opening pressure of 12 cm/H2O. Cerebrospinal fluid (CSF) analysis revealed a cell count of 20, with 80% lymphocytes and 20% monocytes. Glucose level was 49, and protein level was 31. CSF cryptococcal antigen was negative, and CSF cultures, including fungal culture, were also negative.

**Figure 3:**
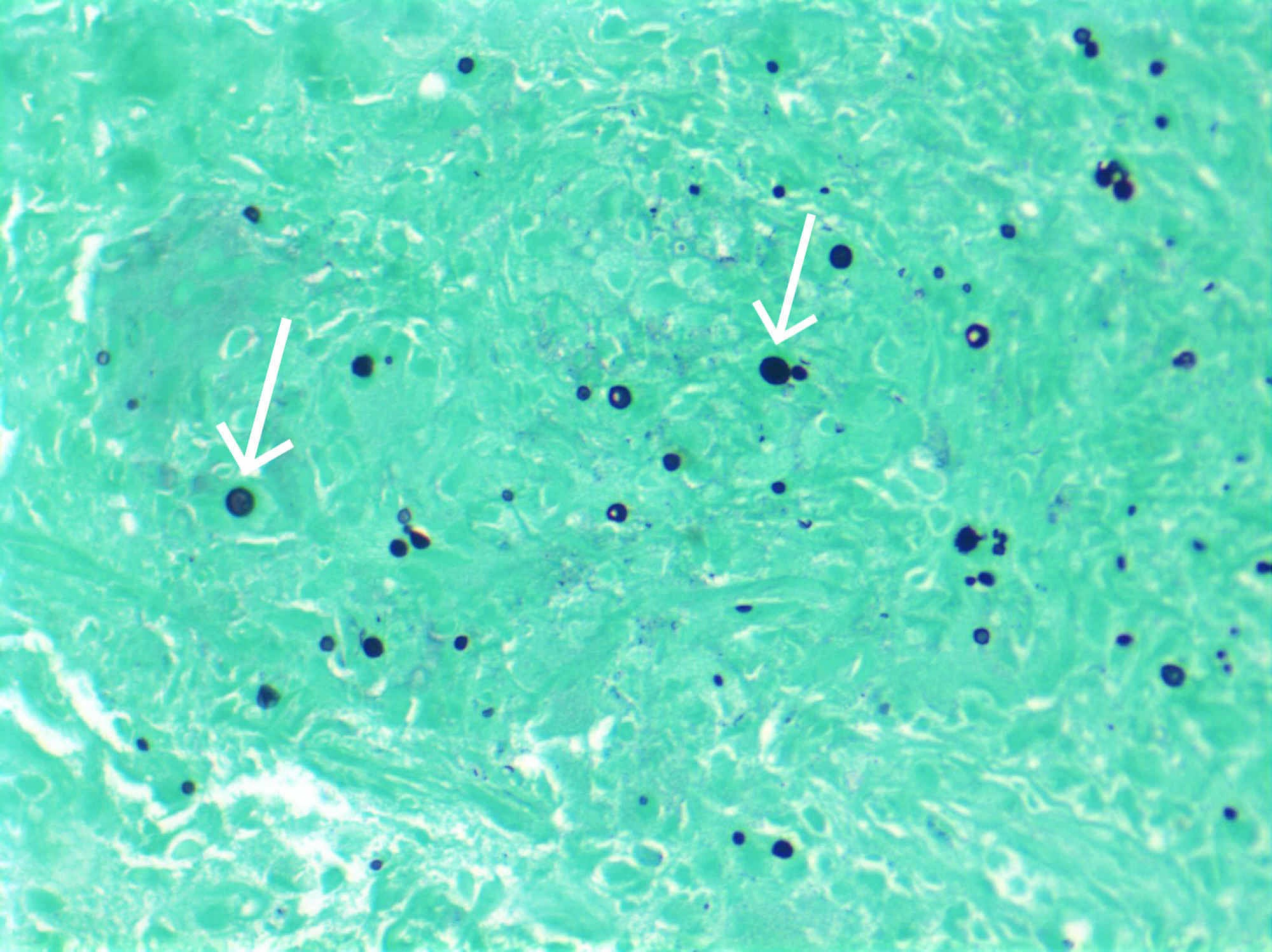
GMS staining of specimen taken from CT-guided biopsy of right upper lobe nodule GMS, Gomori Methenamine-Silver; CT, computed tomography

At this stage of the workup, it was unclear where the cryptococcal infection was located. There was no other evidence of disseminated disease, with negative results from the LP and no apparent lesions on skin examination. In order to confirm the suspected diagnosis of pulmonary cryptococcoma, the patient underwent CT-guided biopsy of the larger RUL nodule. Pathology revealed round structures with asymmetric budding on Periodic acid-Schiff-diastase (PAS-D) and Grocott methenamine silver (GMS) stains (Figure 3).

**Figure 4:**
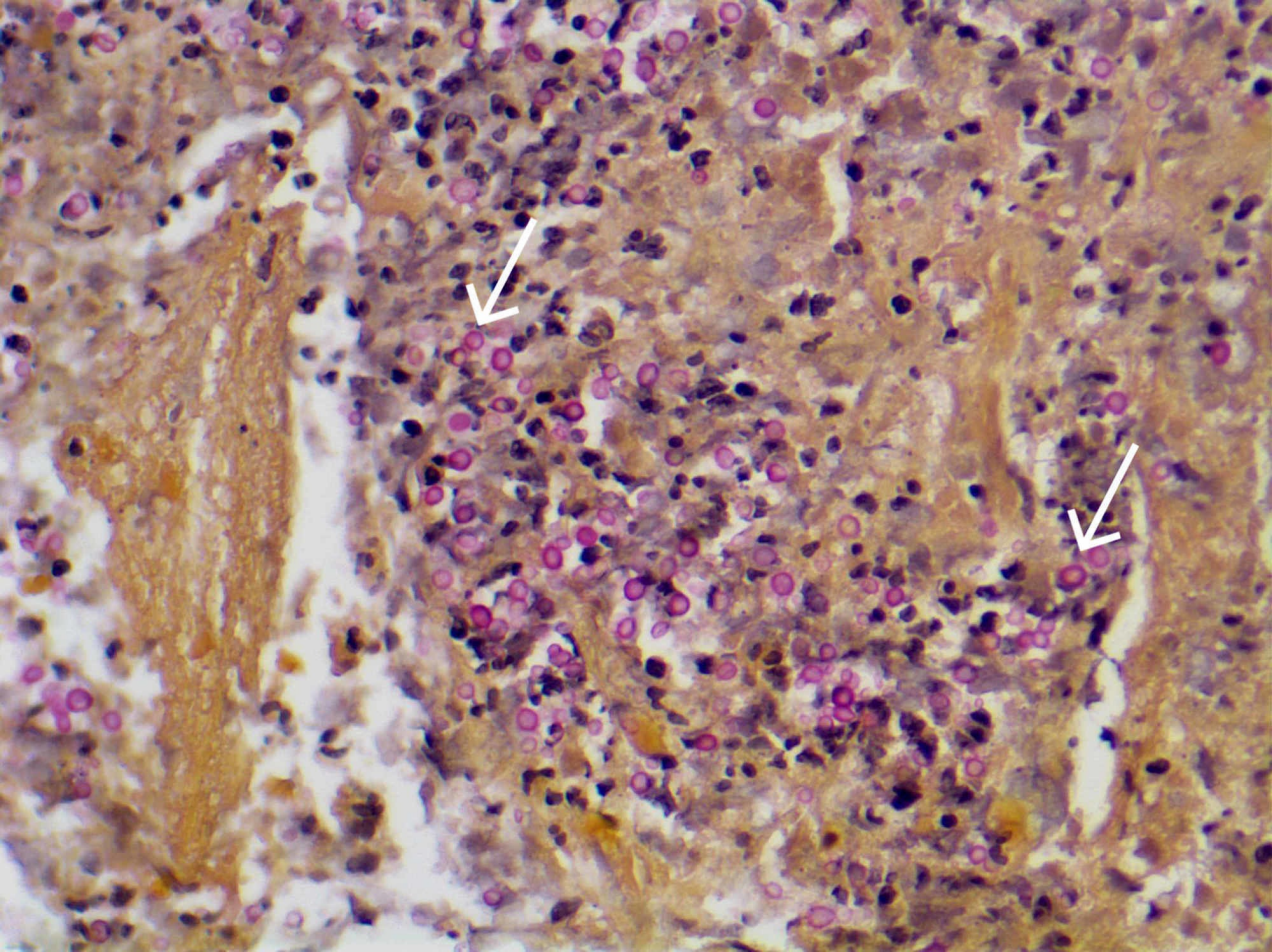
Mucicarmine staining of specimen taken from CT-guided biopsy of right upper lobe nodule CT, computed tomography

There were also round structures on the mucicarmine stain, morphologically most compatible with *Cryptococcus* species (Figure 4).

However, fungal cultures of sputum, blood, and CSF remained negative. As no CNS involvement was evident, the patient's antifungal regimen was de-escalated from liposomal amphotericin and flucytosine to oral fluconazole, with a plan to complete a six to twelve month course of therapy. The ultimate duration would depend on the patient’s recovery of her CD4 count. From our literature review, there have been fewer than forty reported cases of isolated pulmonary cryptococcosis in the setting of immunocompromise (Table 1).

**Table 1:** Literature review of cases where cryptococcosis presented as an isolated pulmonary disease in immunocompromised individuals

Authors & Year	Number of patients with isolated pulmonary cryptococcosis	Serum cryptococcal antigen positivity	Total number of patients included in results of study	Type of Immunocompromise	Age (years)	Sex	Treatment	Mortality rate amongst those with isolated pulmonary cryptococcosis
Cameron et al. 1990	1	Unknown	12	HIV/AIDS	Unknown	Unknown	Unknown	Unknown
Singh et al. 2008	30	Positive in 22/30	48	Organ Transplant	Median: 53.2	36 male, 12 female	Unknown	0.03%
Tarai et al. 2010	1	Positive	1	Renal transplant	65	Male	Liposomal amphoterecin and flucytosine, unknown duration	0%

## Discussion

This case demonstrates that cryptococcal disease may still be localized despite having a positive serum cryptococcal antigen. This is a rare occurrence which illustrates that cryptococcoma should be on the differential when pulmonary lesions are found, especially in combination with a positive cryptococcal antigen.

Cryptococcosis is an important fungal infection that is usually found in immunocompromised patients such as those with advanced HIV, those who have had organ transplants, or those who take chronic steroids or other immunosuppressive medications. It also occasionally develops in immunocompetent hosts who have a lymphocytic dysfunction or an idiopathically low CD4 count [1]. The two most common causative organisms of human disease are *Cryptococcus neoformans* and *Cryptococcus gattii*, which are present worldwide [2]. It has been shown that *C. gattii* infections are associated with healthy individuals while the majority of *C. neoformans* infections develop in immunocompromised patients and are associated with higher mortality [3]. The infection occurs when the host inhales spores found in dust, decayed wood, and bird droppings, which in turn triggers granulomatous inflammatory responses [4,5]. The yeast is then able to invade the bloodstream and travel to other organ sites. Most commonly, cryptococcosis manifests as a CNS issue, such as meningitis, meningoencephalitis, or cerebral cryptococcoma [1]. However, it can also present as a pulmonary disease, which is typically associated with having already developed widely disseminated disease [6-7]. This localized pulmonary presentation is rarely seen in isolation amongst immunocompromised individuals and hence is less readily diagnosed. Other diseases may take precedence when forming a differential for pulmonary cryptococcosis, including malignancy, pneumonia, pulmonary tuberculosis, and other granulomatous or opportunistic fungal infections [2,5]. One infection that may present very similarly in AIDS patients is PCP [6-7]. Symptoms can include fever, shortness of breath, cough, or chest pain [8-9]. The clinical features of pulmonary cryptococcosis may range from no symptoms at all to life-threatening conditions, such as ARDS and septic shock. In many patients, the infection may lay dormant for years before a trigger causes reactivation to overt disease [8-9].

The diagnosis of pulmonary cryptococcosis is made based on serum cryptococcal antigen, fungal culture, chest imaging, as well as histopathologic examination [2,6]. The serum cryptococcal antigen is a highly sensitive test. It can be detected prior to the onset of symptoms, and high titers usually indicate advanced disease [2,4,6]. However, it is rarely positive in patients who do not have disseminated disease [2]. In HIV-infected patients with serum antigen titer ≥1:512, empiric treatment for CNS disease is recommended, even if they are asymptomatic [6]. In our case, the serum cryptococcal antigen was positive despite no findings of disseminated disease.

Cryptococcal antigen can be ordered on respiratory fluid obtained via bronchoscopic alveolar lavage (BAL); however, dilution of BAL fluid can yield false-negative results. Respiratory fungal culture has low sensitivity, and false-positive results are common in colonized patients [2]. Imaging findings are most commonly lung nodules, and the spectrum of nodules differs based on the duration of the infection and the host defense mechanisms. They are typically non-cavitary, non-calcified, well-circumscribed lesions and may be single or multiple. They are more commonly beneath the pleura and in the inferior lobes, with sizes varying from 0.5-4 cm. In immunocompromised hosts, pulmonary cryptococcosis tends to present with alveolar and/or interstitial opacities, however, granulomas, cavitary lesions, and organizing pneumonia may also be seen [1,10-11]. Histologic examination of fluid obtained via BAL and staining with India ink, mucicarmine, periodic-acid Schiff, alcian blue stain, or Grocott-Gomori can demonstrate the fungus and confirm the diagnosis [1]. Histopathologic examination is also required to confirm the suspected disease in other sites such as skin or lymph nodes [6]. Transbronchial lung biopsy with staining is typically not necessary for diagnosis [12].

Patients with suspected pulmonary cryptococcosis should undergo an evaluation with BAL or sputum culture for *C. neoformans*. In addition, they should be evaluated for disseminated disease by obtaining blood cultures and serum cryptococcal antigen. More importantly, lumbar puncture should be performed for CSF culture and CSF cryptococcal antigen, in order to rule out CNS disease. This ultimately affects the duration of treatment and monitoring [8]. The LP typically yields a high protein, high white cell count, and high opening pressure [13]. The high opening pressure is attributed to the failure of CSF resorption via the arachnoid villa due to the physical obstruction by the cryptococcal polysaccharide capsules [14].

Of note, testing CSF cryptococcal antigen can also yield false-negative results, hence disseminated infection with CNS involvement cannot be ruled out [15]. Low CD4 counts are associated with cryptococcosis. In one case review, CD4 counts were found to be low in all cases of pulmonary cryptococcosis, and overall survival time was poor, even in patients who received treatment. Higher CD4 counts and lower cryptococcal antigen titers were found in those with isolated pulmonary cryptococcosis compared with those who had disseminated disease [9].

Once the diagnosis of mild to moderate isolated pulmonary cryptococcosis is confirmed, patients are treated with fluconazole 400 mg by mouth daily for a total of 6 to 12 months, with the aim of resolution of symptoms and prevention of dissemination to the CNS. In some occasions, cryptococcosis may manifest as ARDS in the setting of Immune Reconstitution Inflammatory Syndrome (IRIS), in which case, corticosteroids may be used [8]. The treatment of disseminated cryptococcosis, severe pulmonary cryptococcosis, or meningeal/cerebral cryptococcosis is the same for both *C. neoformans* and *C. gattii*; however, a longer duration is recommended for the latter. Induction treatment with a combination of amphotericin B and flucytosine is given for two weeks. Monitoring for nephrotoxicity is necessary during treatment with amphotericin B [6]. Consolidation treatment with fluconazole monotherapy is recommended for at least eight weeks. There have been recent studies reporting resistance of the organism to fluconazole; however, antifungal susceptibility is not routinely tested. Some small studies have reported that the higher the MICs to antifungals, the worse the treatment outcomes. After the consolidation phase, patients are continued on maintenance treatment with a reduced dose of fluconazole and may be stopped after one year if CD4 counts in HIV patients on ART therapy improve to >100 cells/µL and cryptococcal antigen titers are <1:512. In some cases, surgery may be an option for those who do not respond to treatment or have radiographic evidence of non-resolving disease [6,8].

Of note, in one study, the mortality rate of cryptococcosis in countries where ART is available was found to be 15-20%, compared to a mortality rate of 32% in countries without readily accessible ART, such as Uganda [16-17]. It is important to note that the use of ART in HIV patients is essential for avoiding relapse of cryptococcal meningitis. If their immune system improves, there would be no further need for secondary prophylaxis for cryptococcal meningitis, which is otherwise given indefinitely [18]. Interestingly, improved technology (e.g. multiplex CSF PCR) has made it easier to rapidly diagnose cryptococcal meningitis, but with cART the incidence has actually decreased [14,19].

In one case report, a patient post-renal transplant who was on immunosuppressive medications was found to have nodular opacities in bilateral lungs and was diagnosed with pulmonary cryptococcosis by BAL culture. He was also found to have a serum positive cryptococcal Ag and was treated with flucytosine and amphotericin B [20]. The discrepancy between treatments in our case versus the one reported above is that our patient did not show severe symptoms of pulmonary disease. Hence, she was classified as having mild to moderate pulmonary cryptococcosis and was appropriately treated with fluconazole alone. Our patient did not have the dissemination of the disease into the blood or CNS, as her blood and CSF cultures were negative. Her symptoms of subjective fever, chills, and productive cough were attributed to the pulmonary nodules, and it is assumed that her disease was discovered in the early stage before disseminating to the CNS or other sites. Interestingly, it has been reported that a non-virulent strain of *C. neoformans* can cause mild CNS infections, which may explain why our patient did not have serious signs and symptoms of dissemination [20].

## Conclusions

It is imperative to thoroughly investigate pulmonary lesions that are found in immunocompromised patients, especially individuals with HIV and a low CD4 count, keeping the possibility of localized or disseminated cryptococcosis high on the differential. Diagnostic modalities such as BAL should be utilized, and histopathologic examination should not be delayed in order to obtain a prompt diagnosis to ensure appropriate treatment and optimal patient outcomes.
